# Development and validation of a high throughput, closed tube method for the determination of haemoglobin alpha gene (*HBA1* and *HBA2*) numbers by gene ratio assay copy enumeration-PCR (GRACE-PCR)

**DOI:** 10.1186/s12881-015-0258-y

**Published:** 2015-12-18

**Authors:** Andrew Turner, Jurgen Sasse, Aniko Varadi

**Affiliations:** Department of Pathology and Laboratory Medicine, Sheikh Khalifa Medical City, Abu Dhabi United Arab Emirates; Department of Biological, Biomedical and Analytical Sciences, Faculty of Health and Applied Sciences, University of the West of England, Bristol, UK

**Keywords:** Alpha thalassaemia, Copy number determination, Gene quantification, qPCR, GRACE-PCR

## Abstract

**Background:**

Deletions of the α-globin genes are the most common genetic abnormalities in the world. Currently multiplex Gap-PCRs are frequently used to identify specific sets of common deletions. However, these assays require significant post-amplification hands on time and cannot be used to identify novel or unexpected deletions. The aim of the current study was to develop a rapid screening test for the detection of all deletions of the α-globin genes that can be integrated into a high volume clinical laboratory workflow.

**Methods:**

A gene ratio assay copy enumeration (GRACE) PCR method was developed by simultaneous amplification of targets in the α-globin genes (*HBA1* and *HBA2*) and the chloride channel voltage sensitive 7 (*CLCN7*) reference gene. A novel application of High Resolution Melting (HRM) analysis then allowed rapid determination of α-globin gene copy numbers. The assay was validated using 105 samples with previously determined and 62 samples with unknown α-globin genotypes.

**Results:**

The GRACE-PCR assay detected abnormal α-globin gene copy numbers in 108 of the 167 samples evaluated. The results were consistent with those from a commercial reverse hybridization assay and no allele drop out was observed.

**Conclusions:**

We have successfully developed and validated a GRACE-PCR screening test for the detection of deletions and duplications of the α-globin genes. The assay is based on copy number determination and has the ability to detect both known and novel deletions of the α-globin genes. It is a closed tube technique; consequently the risk of amplicon contamination is negligible. Amplification, detection and analysis can be completed within one hour, making it faster, cheaper and simpler than other existing tests and thus well suited as a rapid first step in a clinical laboratory workflow.

**Electronic supplementary material:**

The online version of this article (doi:10.1186/s12881-015-0258-y) contains supplementary material, which is available to authorized users.

## Background

Deletions of the α-globin genes are the most common genetic disorders in the world. The World Health Organization (WHO) estimates that 20 % of the world’s population may have a deletion in one or more α-globin genes [1]. The majority of these cases are asymptomatic carriers, where the deletion of one or two α-globin genes may provide some protection against malaria [1, 2]. However, deletion of three genes causes a form of thalassaemia intermedia known as Hb H disease and often results in significant anaemia. Deletion of all four α-globin genes leads to Hb Barts hydrops foetalis syndrome and normally results in foetal death; such pregnancies are also associated with a significant risk of maternal morbidity or mortality [3]. It has been estimated that around 13,000 pregnancies are affected annually by severe forms of α-thalassaemia [1].

**Fig. 1:**
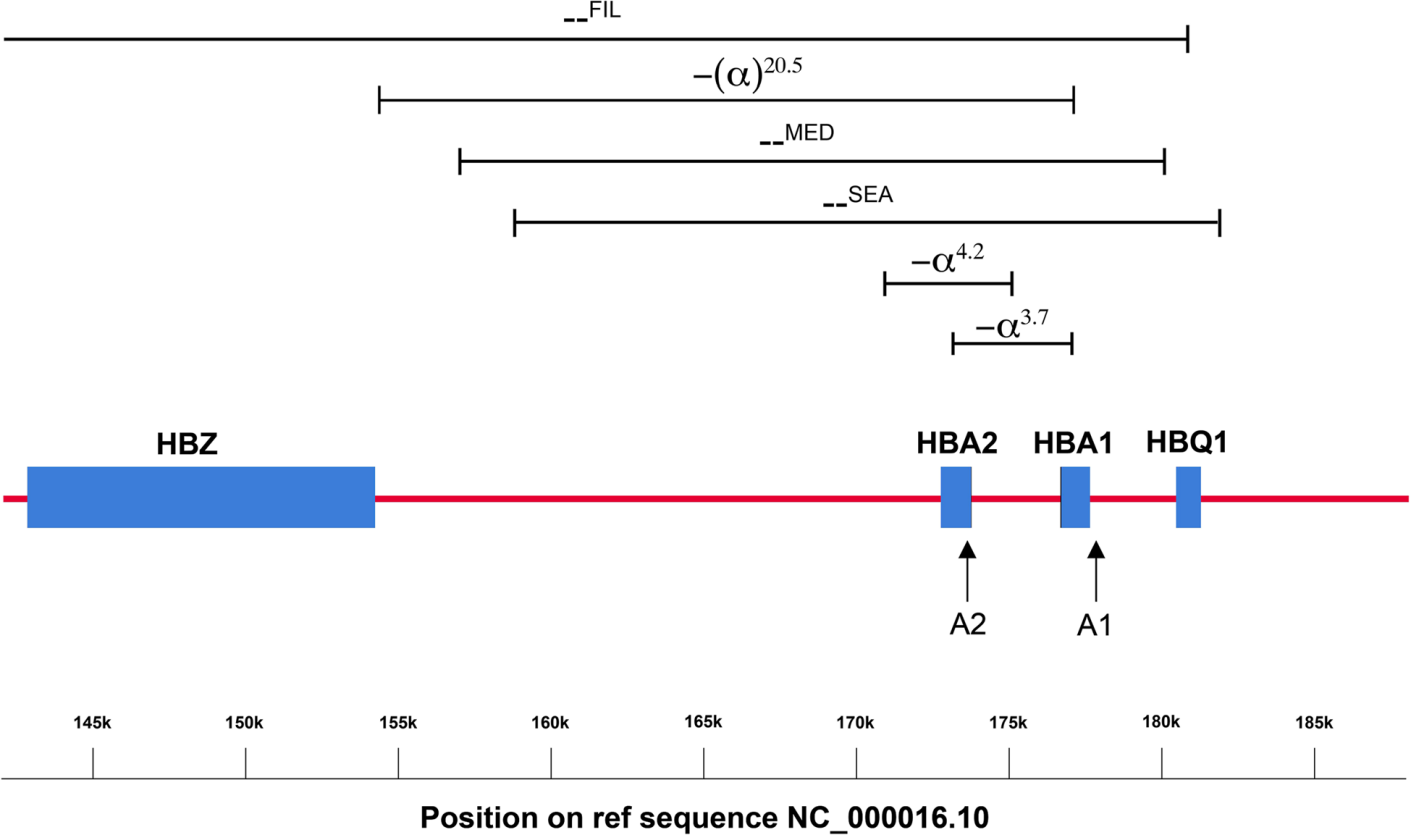
A schematic representation of the α-globin locus from the Haemoglobin Zeta (HBZ) to the Haemoglobin Theta 1 (HBQ1) gene. The approximate position of the gene deletions detected in the current study is shown. The location of amplicons targeting the HBA1 and HBA2 genes are indicated by the arrows A1 and A2, respectively. The CLCN7 gene is located approximately 1.3 million bases 3′ of the α-globin locus (not shown)

Normal haemoglobin is a tetramer composed of two α-globin and two β-globin chains. There are two functional α-globin genes, designated Haemoglobin Alpha 1 (*HBA1*) and Haemoglobin Alpha 2 (*HBA2*). These genes are 97 % homologous and are located 2941 bp apart on chromosome 16 in a region known as the α-globin locus (Fig. 1) [4]. In contrast to β-thalassaemia, where deletions are relatively rare, the majority of α-thalassaemia cases arise from gene deletions [2]. The designation α^+^ is used to describe a chromosome with reduced α-globin gene expression, while α^o^ describes a chromosome with no gene expression [4]. Typically, α^+^ thalassaemia alleles result from a small deletion that leaves just one functional α-globin gene on the chromosome, although there are also non-deletional forms that arise as the result of a point mutation. α^o^ Thalassaemia alleles are normally caused by larger deletions affecting both the *HBA1* and *HBA2* genes [2].

Identification of α-globin gene deletions is often performed in a clinical laboratory setting using either Gap-PCR or reverse hybridization. A number of multiplexed assays based on these techniques have been described for the most common deletions [5–9]. These techniques are simple and fairly robust, but the use of either agarose gel electrophoresis or hybridization for identification makes them relatively time consuming and labour intensive. Thus, these technologies are not particularly convenient for high throughput testing in a routine clinical laboratory. An additional limitation of these techniques is that they only identify specific targeted deletions and rare or novel deletions may be missed.

Due to these limitations, methods for the detection α-globin gene deletions based on a number of alternative techniques have been developed. These include Multiplex Ligation-dependent Probe Amplification (MLPA) [10, 11], Denaturing High Performance Liquid Chromatography (DHPLC) [12, 13], Quantitative PCR (qPCR) [14–18] and melting curve analysis [19–21]. Melting curve analysis methods are particularly attractive, since they do not require post amplification processing by electrophoresis or chromatography. A novel technique based on co-amplification of a target and a reference gene, followed by melting analysis has been described for the Adenomatous Polyposis Coli (*APC*) gene [22]. More recently this technique has also been applied to the Ataxia telangiectasia mutated serine/threonine kinase (*ATM*) and the Phosphate and tensin homolog (*PTEN*) genes [23]. Here we describe the use of a similar approach to develop a novel gene ratio assay copy enumeration (GRACE) screening test for the simultaneous copy number determination of the *HBA1* and *HBA2* genes relative to the reference gene chloride channel voltage sensitive 7 (*CLCN7*) [14]. This GRACE-PCR screening test is a single tube assay that can detect any rearrangements of the α-globin genes that affect the 3′ ends of the *HBA1* or *HBA2* genes. In contrast to most previously described assays for deletion or duplication of the α-globin genes, the GRACE-PCR assay is a simple closed tube technique, which requires no further hands on time after the initial PCR setup. Data analysis is performed by a novel application of the standard features of the HRM analysis software, making it high throughput and well suited for performing rapid initial screening of samples in the clinical laboratory.

## Methods

### Samples

This study was conducted using anonymous, archived material from blood specimens submitted to the Sheikh Khalifa Medical City (SKMC) laboratory, Abu Dhabi, United Arab Emirates for α-thalassaemia screening. Ethical clearance was obtained from the SKMC Institutional Research and Ethics Committee to use this material for the current study. In total 167 samples were used.

The performance characteristics of the GRACE-PCR assay were originally established using 105 samples with known genotype. These samples were selected to represent as many different α-globin genotypes as possible. Samples with point mutations of the *HBA2* gene in the stop codon or untranscribed region (UTR) were also included to assess their impact on assay performance. The assay was further validated using an additional 62 samples of unknown genotype.

### DNA Extraction

Genomic DNA was extracted from whole blood using the QIAamp DNA Blood Mini kit (Qiagen, Germany) in accordance with the Manufacturer’s instructions. The concentration of the extracted DNA was measured using a NanoDrop 2000 spectrophotometer (Thermo Scientific, USA) and adjusted to 10 ng/μl with 10 mM Tris, 0.5 mM EDTA (pH 9.0).

### Genotyping

All samples included in the study were genotyped using the CE-IVD (Conformité Européene - In Vitro Diagnostics) marked α-Globin StripAssay (ViennaLab Diagnostics, Austria) in accordance with the manufacturer’s instructions. The StripAssay is able to detect the ααα^anti3.7^ duplication and the following deletions: -α^3.7^, -α^4.2^, -(α)^20.5^, --^SEA^, --^Med^, --^Fil^, --^Thai^ in addition to a number of point mutations including α^Constant Spring^α, α^Icaria^α, α^polyA-1^α and α^polyA-2^α.

### Primer design and synthesis

To detect deletions in *HBA1* and *HBA2* simultaneously three primer pairs targeting the *HBA1*, *HBA2* and *CLCN7* genes were used in the GRACE-PCR assay (Table 1). All primers were designed using Oligo Primer Analysis Software version 7.56 (Molecular Insights, USA). The approximate location of the amplification sites for the *HBA1* and *HBA2* genes along with the locations of the deletions identified in the current study are shown in Fig. 1. Primers were specifically designed to work at the same annealing temperatures, but to generate products with melting temperatures approximately 3 °C apart. Alternative primer pairs were also developed to amplify *HBA2* and *CLCN7* genes by GRACE-PCR to investigate anomalous results from the original GRACE-PCR (Additional file 1: Table S1).

**Table 1:** Primers used for the GRACE-PCR alpha globin copy number assay

Primer Direction	Primer Sequence 5′-3′	Target Gene symbol	Primer Concentration (μM)	Product size (bp)	Product Tm (°C)	Position on Ref Sequence NC_000016.10
Forward	CACCCGGCCTCATGGAT	*CLCN7*	0.16	155	79.4	1,462,101 to 1,462,255
Reverse	AAGAGAACTACAGACCAACACCC					
Forward	CCATCTTTACGTTTCTGGGCACTC	*HBA1*	0.45	131	82.2	177,800 to 177,930
Reverse	GCCATGCTGGAGTGGGACTTC					
Forward	CCGTTAAGCTGGAGCCTCGGT*A	*HBA2*	0.45	171	85.2	173,594 to 173,764
Reverse	ACACCTCCATTGTTGGCACAT					

*indicates the use of phosphorothioate (PTO) to block 3′ exonuclease activity

High Performance Liquid Chromatograph (HPLC) purified primers were commercially synthesized (Metabion, Germany).

### GRACE-PCR and data analysis

Each 12.5 μL GRACE-PCR reaction contained 20 ng of genomic DNA, 0.25 units of Kappa HiFi HotStart polymerase (Kappa Biosystems, Boston, USA), 0.25 units of Platinum Taq polymerase (Invitrogen, Carlsbad, USA), 0.5 μL of LightCycler 480 ResoLight dye (Roche Diagnostics, Mannheim, Germany), 1x Kappa GC buffer (Kappa Biosystems), 0.3 mM of each dNTP (Kappa Biosystems), 0.45 μM of each primer for the *HBA1* and *HBA2* genes and 0.16 μM of each primer for the *CLCN7* gene.

PCR was performed using a Rotor-Gene Q-5plex HRM thermocycler (Qiagen, Germany). This system can accommodate up to 72 PCR reactions per run. After an initial 5 min hold at 95 °C, 27 cycles of PCR were performed as follows: 10 s at 98 °C, 10 s at 58 °C and 10 s at 72 °C. Melting was performed at a rate of 0.2 °C/s over the temperature range 77 °C to 87 °C, with data acquisition on the HRM channel. PCR conditions for the alternative primers are given in the Additional file 1: Method section.

Data analysis was performed using the HRM module Rotor-Gene Q series software version 1.7.

## Results

### GRACE-PCR design and optimisation

**Fig. 2:**
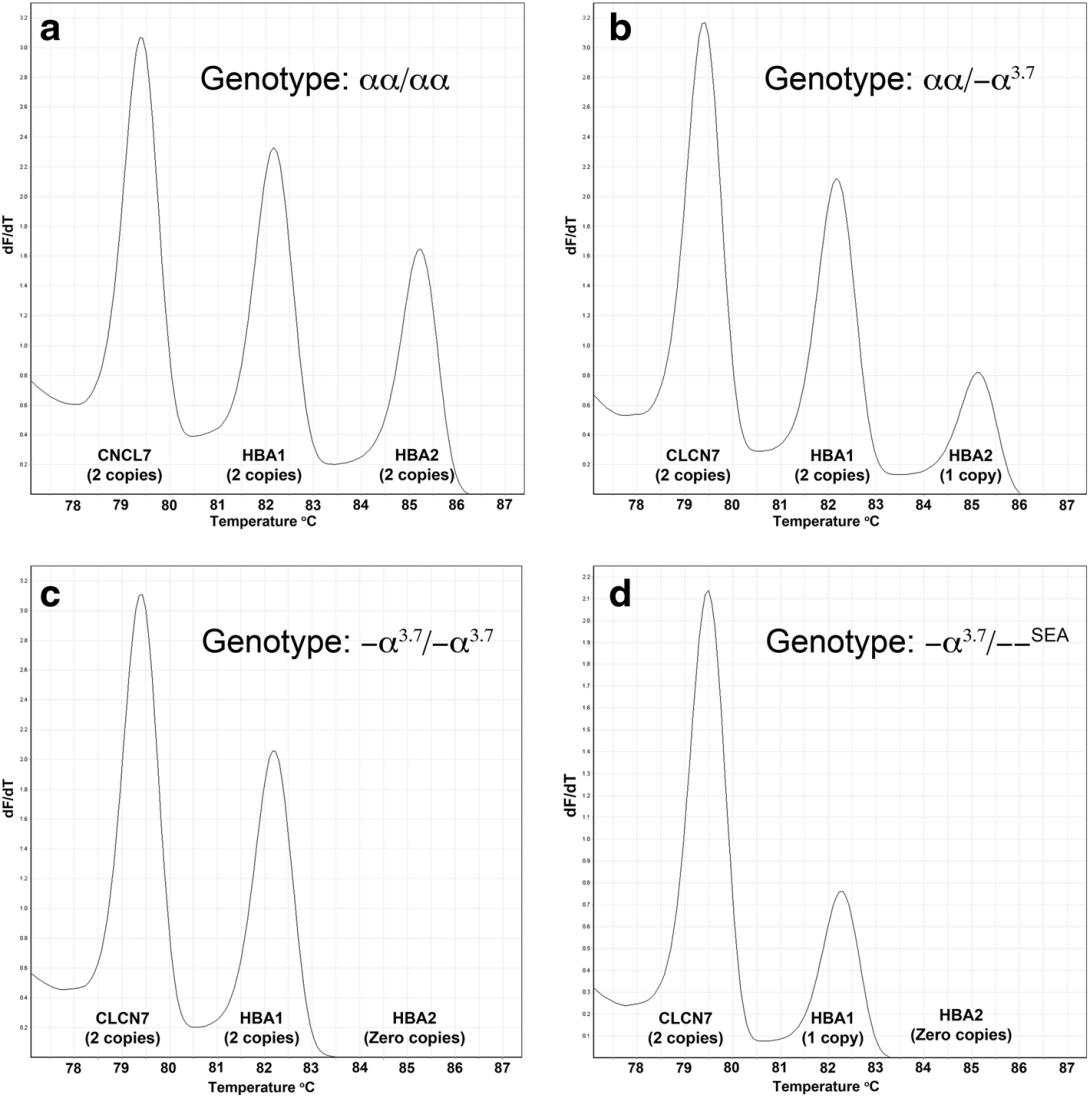
GRACE-PCR assay, limited cycle multiplex PCR targeting both α-globin genes (*HBA1* and *HBA2*) and a reference gene (*CNCN7*). Peak height, relative to the reference gene, in the melting plots (−dF/dT versus temperature) is proportional to the copy number of each gene (indicated in parenthesis under the gene name). Four genotypes with varying α-globin gene copy numbers are shown in plots **a** to **d**

Initially, we used Platinum Taq polymerase in combination with 1.2 M betaine during the development of the GRACE-PCR assay. This strongly favoured the amplification of *HBA2* over *HBA1*, which made the quantification of the latter difficult. This issue could not be resolved by a simple redesign of the primers. Consequently, various alternative enzymes and enhancers were tried and with the use of an enzyme cocktail containing Platinum Taq and Kappa HiFi HotStart polymerases simultaneous and comparable amplification of *HBA1* and *HBA2* was achieved (Fig. 2). The Kappa HiFi HotStart polymerase has 3′ to 5′ exonuclease proofreading activity, thus to avoid mispriming of the *HBA2* forward primer to *HBA1* a phosphorothioate (PTO) block was incorporated in the primer sequence (Table 1) [24]. Amplification of the *CLCN7* control gene [14, 25] was more efficient than that of the target α-globin genes, which was corrected by reducing the concentration of the *CLCN7* primers (Table 1). Limiting the number of PCR cycles resulted in termination of amplification during the exponential phase of the PCR reaction. This ensured that the relative amount of each PCR product remained proportional to the initial number of copies of the gene present.

### Data analysis

**Fig. 3:**
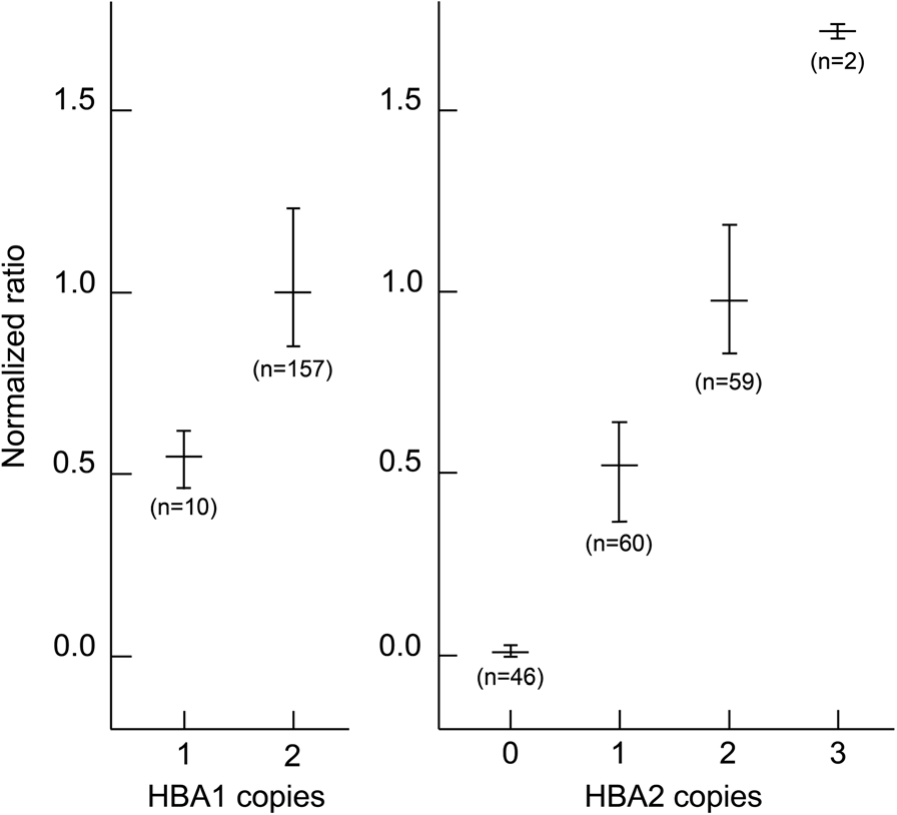
Normalized ratios of -dF/dT peak heights for different copy numbers of the *HBA1* and *HBA2* genes. The horizontal bars indicate the mean ratio and the vertical bars indicate the range from the minimum to maximum ratio observed for each gene copy number. The data was normalized with the equation: Normalized ratio = (R_WT_ _Control_/T_WT_ _Control_)/(R_Sample_/T_Sample_), where R and T is reference gene and target gene peak heights, respectively

**Fig. 4:**
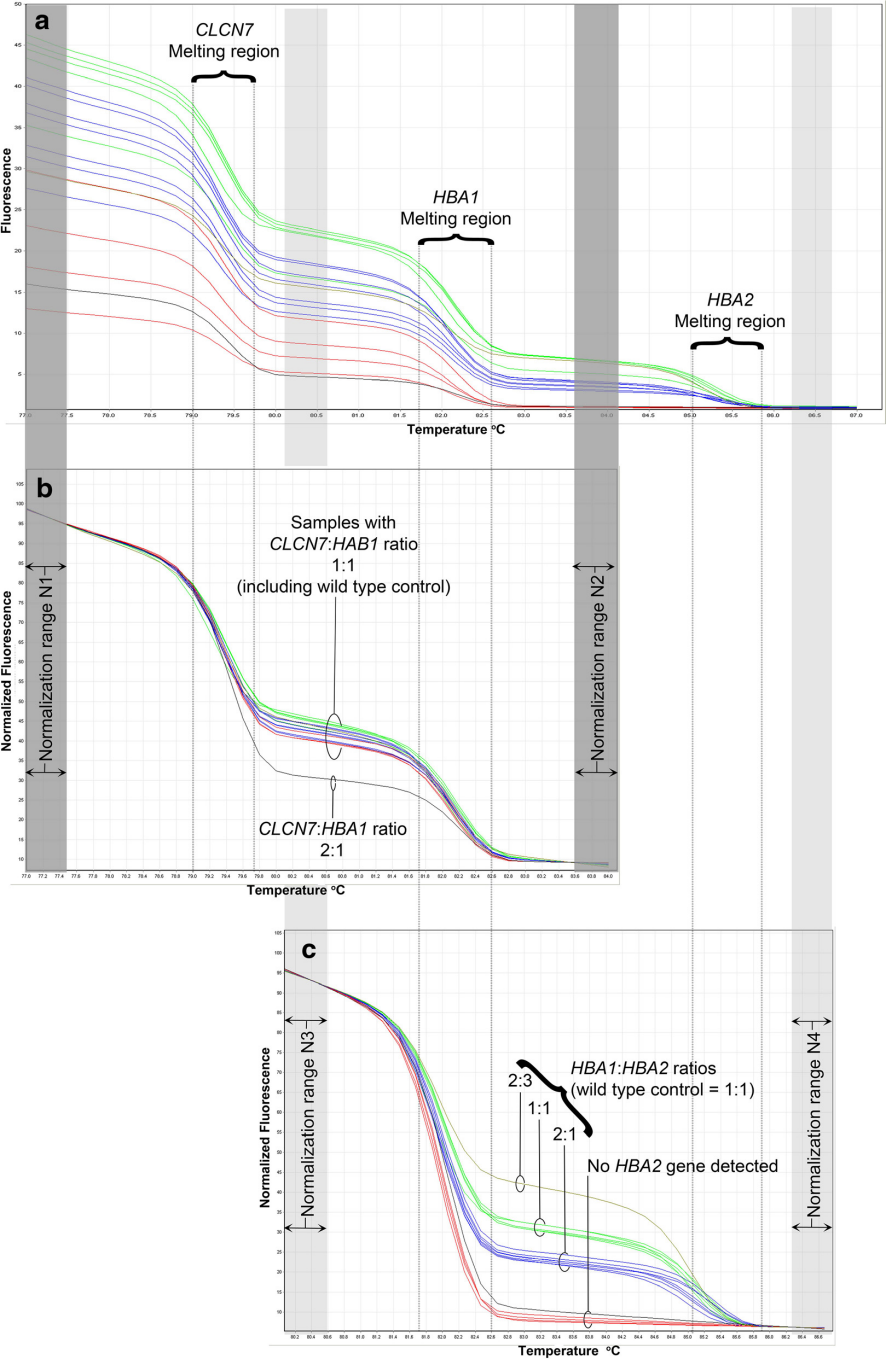
Normalization of GRACE-PCR assay to determine gene copy numbers. Raw melting curves show three distinct steps corresponding to the melting of the *CLCN7, HBA1* and *HBA2* gene products generated during the GRACE-PCR Screening Test (**a**). Setting the normalization regions N1 and N2 (dark grey bars) in the HRM analysis software allowed the gene copy number ratio for *CLCN7*:*HBA1* to be determined (**b**). Re-setting the normalization regions to N3 and N4 (light grey bars) allowed the determination of the gene copy number ratio *HBA1:HBA2* (**c**). The copy number for *CLCN7* is assumed to be two, which allows the number of *HBA1* copies to be calculated, which in turn allows the determination of the number of *HBA2* copies

The amount of PCR product from each of the three genes targeted in the GRACE-PCR assay is proportional to the height of the peak on the -dF/dT versus temperature plots (Fig. 2). Initially we determined the gene copy numbers by measuring peak heights on the -dF/dT plots (Fig. 2) and then normalized the data to a wild type control sample included in the same run using the following calculation: Normalized ratio = (R_WT Control_/T_WT__Control_)/(R_Unknown_/T_Unknown_); where R and T are the peak heights for the reference (*CLCN7*) and the target (*HBA1* or *HBA2*) genes, respectively. The subscript ‘WT Control’ indicates the wild type control sample. This process worked well and allowed the correct copy number for both α-globin genes to be determined for all samples tested (Fig. 3). However, this method of data analysis was complex and time consuming. Consequently, we developed a simplified visual method for data analysis, which utilized features available in the HRM module Rotor-Gene Q series software (Fig. 4) and eliminated the need for manual calculations.

As expected in the GRACE-PCR assay three distinct melt transition steps could be observed in the melting curves corresponding to the melting temperatures of the PCR products initially designed to be 3°C apart (Fig. 4a, Table 1). Therefore, the steps could be assigned to the melting of the products from *CLCN7, HBA1* and *HBA2* genes as indicated in Fig. 4a. Normalization of the raw HRM data was performed in two stages using the Rotor-Gene Q series software. First the data was normalized by setting normalization ranges before the melting of the *CLCN7* product and after the melting of the *HBA1* product (normalization ranges N1 and N2 indicated by the dark grey bars in Fig. 4). This resulted in a plot in which the height of the curve between the *CLCN7* and *HBA1* melt regions is dependent on the gene copy number ratio of *CLCN7*:*HBA1*. Inclusion of a known wild type control sample in each run allowed rapid visual determination of any samples with abnormal copy numbers for *HBA1* (Fig. 4b). Since the number of copies of the *CLCN7* reference gene is assumed to be two, this ratio can be used to determine the copy number for the *HBA1* gene. In the second stage of the analysis normalization ranges were set before the melting of the *HBA1* product and after the melting of the *HBA2* product (using the normalization ranges N3 and N4 indicated by the light grey bars in Fig. 4c). This resulted in a plot allowing the gene copy number ratio of *HBA1:HBA2* to be determined (Fig. 4c). Since the copy number for the *HBA1* gene was already determined in the first stage of the analysis this ratio allowed the copy number for *HBA2* to be easily derived. This alternative visual method of analysis led to identical results as the more complex calculation method for all 167 samples included in the study, but proved far quicker and simpler to perform.

### Assessment and validation of the GRACE-PCR assay

An initial assessment of the assay was carried out using 105 samples that had been previously genotyped with the commercial α-Globin StripAssay (ViennaLab Diagnostics, Austria). These 105 samples included 70 samples that had deletions or duplications of one or more of the α-globin genes. Samples with mutations of the *HBA2* stop codon and UTR were also included to ensure that they did not interfere with the assay performance.

GRACE-PCR correctly identified the 70 samples shown by the α-globin StripAssay to have deletions or duplications of the α-globin genes (Table 2). Mutations of the stop codon or UTR did not affect the copy number result, however, the -(α)^20.5^ deletion (Fig. 1) was detected as a deletion of the *HBA2* gene only. This was because the -(α)^20.5^ deletion removes all of the *HBA2* gene and the 5′ end of the *HBA1* gene, but does not extend to the primer target at the 3′ end of the *HBA1* gene. The performance of the GRACE-PCR assay was further validated by the analysis of an additional 62 samples prior to genotyping by the α-globin StripAssay. GRACE-PCR detected α-globin gene deletions in 38 of these samples, all of which were subsequently confirmed by genotyping (Table 2). Additionally, 143 samples were analysed using alternative primers (Additional file 1: Table S2) and no allele drop out was observed.

**Table 2:** Genotypes of samples used in the development and validation of the alpha globin gene ratio analysis copy enumeration PCR assay (GRACE-PCR)

α-Globin StripAssay		GRACE-PCR Screening Test					
Genotype	Strip Assay (n)	PCR 1 (n)	PCR 2 (n)	*CLCN7:HBA1* Ratio	*HBA1:HBA2* Ratio	*HBA1* Copies	*HBA2* Copies
1. αα/αα (wild type)	54	30	24	1:1	1:1	2	2
2. αα/-α^3.7^	48	29	19	1:1	2:1	2 ^(a)^	1
3. -α^3.7^/-α^3.7^	36	20	16	1:1	1:0	2 ^(a)^	0
4. αα/-α^4.2^	2	2	0	1:1	2:1	2	1
5. -α^4.2^/-α^4.2^	1	1	0	1:1	1:0	2	0
6. -α^3.7^/-α^4.2^	3	3	0	1:1	1:0	2 ^(a)^	0
7. -α^3.7^/--^SEA^	6	3	3	2:1	1:0	1 ^(a)^	0
8. αα/--^Med^	2	2	0	2:1	1:1	1	1
9. αα/--^Fil^	2	2	0	2:1	1:1	1	1
10. -(α)^20.5^	1	1	0	1:1	2:1	2 ^(b)^	1
11. αα/ααα^anti3.7^	2	2	0	1:1	2:3	2	3
12. -α^3.7^/α^Icaria^α ^(c)^	1	1	0	1:1	2:1	2 ^(a)^	1
13. αα/α^poly-A1^α ^(c)^	2	2	0	1:1	1:1	2	2
14. αα/α^poly-A2^α ^(c)^	1	1	0	1:1	1:1	2	2
15. -α^3.7^/α^polyA-1^α ^(c)^	4	4	0	1:1	2:1	2 ^(a)^	1
16. αα/α^Constant Spring^α ^(c)^	1	1	0	1:1	1:1	2	2
17. α^Constant Spring^α/α^Constant Spring^α ^(c)^	1	1	0	1:1	1:1	2	2
Total number of samples	167	105	62				

The GRACE-PCR assay was assessed using samples with previously determined (PCR1) and unknown (PCR 2) genotypes. GRACE-PCR was able to detect all 108 samples identified by the commercial α-globin StripAssay as having α-globin gene rearrangements (genotypes 2 to12)

(a) Due to the positioning of primers at the 3′ ends of the α-globin genes, the hybrid -α^3.7^ gene is counted as an *HBA1* gene in this assay

(b) The -(α)^20.5^ deletion does not extend to the 3′ end on the *HBA1* gene targeted by the GRACE-PCR screening test primers, consequently only the deletion of the *HBA2* gene is detected

(c) Samples with point mutations (genotypes 12 to 17) were included to ensure that these point mutations did not affect the gene-copy number determination of the GRACE-PCR assay

In total 108 of the samples were identified by the α-globin StripAssay as having a deletion or duplication of the α-globin genes. GRACE-PCR returned abnormal α-globin gene copy numbers for all 108 positive samples and did not generate any false positives (Table 2). This equates to a sensitivity of 100.0 % (95 % confidence interval 96.6-100.0 %) and a specificity of 100.0 % (95 % confidence interval 93.9-100.0 %).

## Discussion

Conventional Gap-PCR and reverse hybridization assays are commonly used methods to detect α-globin gene deletions [5–9]. These techniques are reliable and allow identification of the specific targeted gene rearrangements. However, Gap-PCR is not ideal for high volume testing in a clinical laboratory, since it is relatively time consuming and labour intensive. PCR protocols for Gap-PCR require a long extension step in order to generate fairly large amplicons; consequently cycling is slow and typically takes approximately 3 h. The subsequent detection by agarose gel electrophoresis involves considerable hands on time, adding around another 2 h to the analysis. In common with Gap-PCR, reverse hybridization assays are open tube techniques that take several hours to complete. Therefore, a stepwise clinical laboratory investigation incorporating a rapid screening step of α-globin gene deletions has been suggested to be a more efficient approach [26]. In our proposed workflow here, all samples are first screened by the rapid and inexpensive GRACE-PCR assay, and then all those identified with a variant copy number are analysed further with a different method that can identify the specific genotype. The initial step in our workflow has the additional advantage that it can detect novel rearrangements not identified by other assays, for example Gap-PCR.

Fragment analysis by capillary electrophoresis have also been used to determine α-globin gene copy numbers [26–29]. These assays make use of the eight base length differences between the *HBA1* and *HBA2* genes. One limitation of these assays is that due to rearrangements of the α-globin genes, such as the African α2 Polymorphism or the α12 allele, this difference in length between the *HBA1* and *HBA2* genes can be absent [30–32]. Indeed, these polymorphisms are reported to be very common, with the African α2 Polymorphism reaching a frequency of up to 12 % in some African populations [30]. In contrast, GRACE-PCR is not affected by these gene rearrangements and it does not require the use of a capillary electrophoresis based genetic analyser making it a more universally applicable and faster screening method.

In common with most PCR based techniques, GRACE-PCR could potentially yield incorrect results due to a SNP within a priming site inhibiting primer annealing and therefore causing allele dropout. The 5′-end of the reverse primer for *HBA2* coincided with the stop codon and consequently, there was a risk that mutations of this codon might result in allele dropout. This was tested by including samples from individuals carrying a mutation in the stop codon, Hb Constant Spring (HBA2:c.427T > C) and Hb Icaria (HBA2:c.427T > A), in the assay validation. Our results showed that the assay design was robust and the presence of the mutations of the *HBA2* stop codon did not affect the results (Table 2, Genotypes 12, 16 and 17).

Some other low frequency SNPs have also been described within the GRACE-PCR priming sites, the most frequent of which (rs2261869) has a minor allele frequency (MAF) of T = 0.0016 [33]. Thus, the possibility of allele dropout needs be considered if an abnormal GRACE-PCR result is obtained that cannot be confirmed by other methods. One possibility for checking the original GRACE-PCR screening result is the use of alternative primers for *HBA2* and *CLCN7* (Additional file 1: Table S1) before proceeding to more complex tests such as MLPA. In the current study all results of the ‘original’ and ‘alternate’ GRACE-PCRs were consistent with the genotype from the StripAssay indicating that no allele dropout had occured, suggesting that such events should be rare.

We also considered the impact that point mutations might have on the performance of GRACE-PCR. Point mutations may alter the melt curve shapes, but would have a negligible impact on total fluorescence, which is dependent on the amplicon amount produced. Consequently, point mutations are not expected to have a significant impact on copy number determination, since GRACE-PCR interpretation is dependent on the fall in fluorescence as each amplicon melts. Indeed, inclusion of samples with the α^Poly A1^ (HBA2:c.*92A > G) and α^Poly A2^ (HBA2:c.*94A > G) mutations in the method validation confirmed that point mutations within the amplicons did not affect the result interpretation (Table 2, Genotypes 13 to 15).

Other melt curve based methods have been previously described for the detection of α-globin gene deletions. Most of these are modified Gap-PCR assays that use melting curve analysis as means of detection, thus eliminating the need for agarose gel electrophoresis [13, 18, 19, 34]. This makes them more convenient than conventional Gap-PCR, but they have only been developed for a limited set of deletions such as --^SEA^ and --^THAI^ [19]. A melt curve method that compares ratios of *HBA1* and *HBA2* gene PCR products has also been described, but this method did not include a reference gene and thus, required additional tubes to detect certain genotypes [20].

The simplicity of quantitative PCR (qPCR) makes it an attractive technology for copy number determination and methods for the α-globin genes have previously been described [14–17]. However, current qPCRs either amplify the reference gene and each target gene in separate tubes [14, 17], or require the use of expensive probes [15, 16]. Thus, such assays are less convenient than the GRACE-PCR screening test, which can be performed in a single tube without the need for probes.

In common with GRACE-PCR, both fragment analysis by capillary electrophoresis and qPCR quantify the α-globin genes relative to a reference gene that is assumed to have a copy number of two. The current GRACE-PCR assay used the well characterized *CLCN7* gene for reference [14, 25, 35]. This gene is well suited for this application because it has no pseudo-genes [14] and mutations lead to autosomal dominant osteopetrosis, an uncommon sclerosing bone disorder [35]. Consequently, individuals without obvious bone abnormalities are expected to have two intact copies of the gene. There is a common SNP (rs2744995, MAF G = 0.3808) at the ultimate 5′ base of the Screening Test *CLCN7* gene reverse primer. Due to its location at the 5′ end of the primer this SNP would not be expected to affect the assay results. Indeed, *CLCN7* copy numbers were identical when alternative primers were used (Additional file 1: Table S2). No information was available on bone abnormalities for the archived material used in the current study, but the genotyping data indicated that all subjects included in the study had two copies of the *CLCN7* gene and neither rs2744995 nor any other SNPs that may have been present affected the results (Table 2). Additionally, in most cases copy number changes or mispriming of the *CLCN7* reference gene would result in an improbable *HBA1* copy number highlighting samples for further investigation.

A limitation of methods that detect gene deletions or duplications by copy number determination is that the presence of deletions or duplications is inferred from the total number of genes, rather than detected directly. When an individual co-inherits both a deletion and duplication of a gene, the total number of genes does not change and consequently such cases would appear normal. For example, the genotype -α^3.7^/ααα^anti3.7^ has the same number of α-globin genes as αα/αα and thus these genotypes cannot be distinguished by copy number. This limitation applies equally to GRACE-PCR, qPCR, capillary electrophoresis fragment analysis and to MLPA. Such combinations would be rare and would not affect the phenotype of the individual carrying the mutations, but they may be of clinical interest in some situations, for example pre-marital genetic counselling.

Copy number based assays do not provide the identity of the specific deletions/duplications detected. However, the GRACE-PCR assay does differentiate between cases with two alleles carrying α^+^ deletions (-α/-α) from cases with a single α^o^ deletion (αα/--). This clinically important distinction can be made because the test uses targets at the 3′ end of each α-globin gene. The advantage of this approach is that the -α^3.7^ hybrid allele types as an *HBA2* gene deletion, thus allowing compound heterozygotes -α^3.7^/-α^4.2^ to be distinguished from a single large α^o^ deletion (αα/--). Assays that utilize targets 5′ of the α-globin genes cannot make this distinction.

Finally, assays based on DHPLC have been described to detect the common -α^3.7^, -α^4.2^ and --^SEA^ deletions [12, 13]. DHPLC requires a specialized chromatography system for detection and the post amplification processing takes around 10 min for each sample. The GRACE-PCR assay has a detection step that is far more rapid, taking around 10 min for a batch of 72 samples. Furthermore, with the use of a plate based PCR system GRACE-PCR batch sizes could be increased up to 384 samples without increasing the post amplification processing time.

## Conclusion

The GRACE-PCR test described here is a simple and robust method for simultaneous copy number assignment of both the *HBA1* and *HBA2* genes. This method is well suited for rapid initial screening of a large number of samples since: (1) unlike Gap-PCR, DHPLC and capillary electrophoresis based methods it does not require extensive post-amplification processing of samples (i.e. electrophoresis or chromatography); (2) in contrast to fragment analysis by capillary electrophoresis-based assays it does not require the use of a capillary electrophoresis based genetic analyser and is not affected by the common African α2 polymorphism; (3) in this closed tube assay the risk of amplicon contamination is negligible; and (4) the data analysis is very simple and allows instant visual identification of any samples in a batch with abnormal α-globin gene copy numbers.

## Additional file

Additional file 1:
**Alternative GRACE-PCR primers. **(DOCX 918 kb)

## Abbreviations

*CLCN7*: Chloride channel voltage sensitive 7 gene
DHPLC: Denaturing high performance liquid chromatography
EDTA: Ethylenediaminetetraaceitc acid
GRACE: Gene ratio assay copy enumeration
*HBA1*: Haemoglobin alpha 1 gene
*HBA2*: Haemoglobin alpha 2 gene
HPLC: High performance liquid chromatography
HRM: High resolution melting
MLPA: Multiplex ligation-dependent probe amplification
PCR: Polymerase chain reaction
PTO: Phosphorothioate
qPCR: Quantitative polymerase chain reaction
WHO: World Health Organization
