# Supplementary material for: Development and validation of a high throughput, closed tube method for the determination of haemoglobin alpha gene (HBA1 and HBA2) numbers by gene ratio assay copy enumeration-PCR (GRACE-PCR)
Source: BMC Med Genet. 2015 Dec 18;16:115. doi: 10.1186/s12881-015-0258-y (PMC4683937; doi:10.1186/s12881-015-0258-y)
Supplement: Additional file 1: — Alternative GRACE-PCR primers. (DOCX 918 kb) [file 12881_2015_258_MOESM1_ESM.docx]

**SUPPLEMENTARY DATA FOR:-**

**Development and validation of a high throughput, closed tube method for the determination of haemoglobin alpha gene (*HBA1* and *HBA2*) numbers by gene ratio assay copy enumeration-PCR (GRACE-PCR)**

**GRACE-PCR with alternative primers to investigate an anomalous result**

Should a situation arise where the GRACE-PCR assay with the original set of primers indicates inconsistent gene copy numbers, and a different technique such as Gap-PCR is not available, repeat analysis by GRACE-PCR with alternative primers is advisable. This approach should be able to confirm if the initial GRACE-PCR result was correct, or if it might have been the result of mispriming.

**METHODS**

Each 12.5 μL PCR reaction contained 25 ng of genomic DNA, 0.25 units of Platinum Taq polymerase, 0.5 μL of LightCycler 480 ResoLight dye, 1x PCR buffer (Invitrogen), 2.0 mM Mg^2+^, 0.3 mM of each dNTP, 1.2 M betaine (Sigma Aldrich, St Louis, MO, USA) and 0.32 μM of each primer (Table 1). After an initial 5 minute hold at 95 ^o^C, 28 cycles of PCR where performed as follows: 5 s at 95 ^o^C, 5 s at 56 ^o^C, 5 s at 72 ^o^C. Melting was performed from 80 ^o^C to 90 ^o^C at a rate of 0.2 ^o^C/s, with data acquisition on the HRM channel.

Data analysis was performed using the High Resolution Melting (HRM) module Rotor-Gene Q series software version 1.7.

**RESULTS**

The use of the alternative primers allowed rapid visual determination of the copy numbers for the *HBA2* gene (Figure 1).

A total of 143 samples were assessed by GRACE-PCR using the alterative primers. For all samples the copy numbers obtained were consistent with results obtained from the original GRACE-PCR assay and the commercial StripAssay (Table 2).

**Table 1: Alternative Primers used for the GRACE-PCR alpha globin copy number assays.**

| **Primer Direction** | **Primer Sequence 5'-3'** | **Target Gene symbol** | **Primer Concentration (μM)** | **Product size (bp)** | **Product Tm (^o^C)** | **Position on Ref Sequence NC_000016.10** |
| --- | --- | --- | --- | --- | --- | --- |
| Forward | ATCAGCCTGTTTCCATAGAACC | *CLCN7* | 0.32 | 194 | 83.2 | 1,473,158 to  1,473,351 |
| Reverse | GCCCCATCACTTCAAATTAACCC |  |  |  |  |  |
| Forward | GCCGTTCCTCCTGCCCGCTG | *HBA2* | 0.32 | 173 | 86.6 | 173,616 to  173,788 |
| Reverse | AGGTCCTTGGTCTGAGACAGGT |  |  |  |  |  |

**Table 2: Comparison of results obtained by GRACE-PCR using both the original and alternative primers.**

| **Genotype**  **(from commercial StripAssay)** | **Samples (n)** | ***HBA2* Copies**  **(Original primers)** | ***HBA2* Copies (Alternative primers)** |
| --- | --- | --- | --- |
| 1. αα/αα (wild type) | 40 | 2 | 2 |
| 2. αα/-α^3.7^ | 48 | 1 | 1 |
| 3. -α^3.7^/-α^3.7^ | 30 | 0 | 0 |
| 4. αα/-α^4.2^ | 2 | 1 | 1 |
| 5. -α^4.2^/-α^4.2^ | 1 | 0 | 0 |
| 6. -α^3.7^/-α^4.2^ | 3 | 0 | 0 |
| 7. -α^3.7^/--^SEA^ | 3 | 0 | 0 |
| 8. αα/--^Med^ | 2 | 1 | 1 |
| 9. αα/--^Fil^ | 2 | 1 | 1 |
| 10. -(α)^20.5^ | 1 | 1 | 1 |
| 11. αα/ααα^anti 3.7^ | 1 | 3 | 3 |
| 12. -α^3.7^/α^Icaria^α | 1 | 1 | 1 |
| 13. αα/α^poly-A1^α | 2 | 2 | 2 |
| 14. αα/α^poly-A2^α | 1 | 2 | 2 |
| 15. -α^3.7^/α^polyA-1^α | 4 | 1 | 1 |
| 16. αα/α^Constant Spring^α | 1 | 2 | 2 |
| 17. α^Constant Spring^α/α^Constant Spring^α | 1 | 2 | 2 |
| Total | 143 |  |  |

The alternative GRACE-PCR primers were tested with 143 samples. For all samples the *HBA2* copy numbers obtained with the alternative primers were consistent with the results obtained using the original GRACE-PCR primers.

**
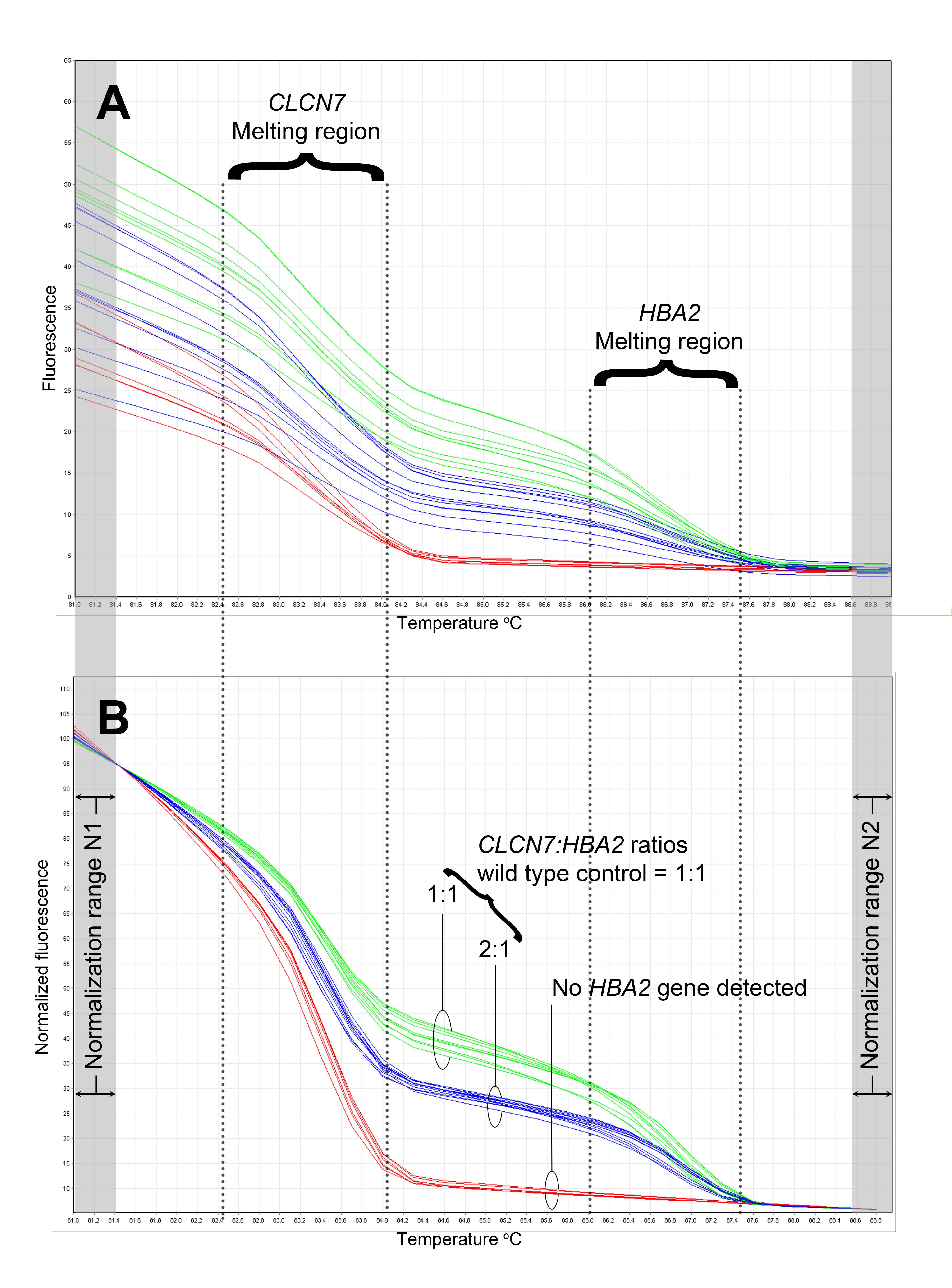
**

**Figure 6: Normalization of the GRACE-PCR data obtained with the alternative primers .**Raw melting curves show two distinct steps corresponding to the melting of the *CLCN7,* and *HBA2* gene products generated during the GRACE-PCR (A). Setting the normalization regions N1 and N2 (grey bars) in the HRM analysis software allowed the gene copy number ratio for *CLCN7*:*HBA2* to be determined (B). The copy number for *CLCN7* is assumed to be two, which allows the number of *HBA2* copies to be calculated (B).
